# Early diagnosis, vertical transmission of HIV and its associated factors among exposed infants after implementation of the Option B+ regime in Ethiopia: a systematic review and meta-analysis

**DOI:** 10.1016/j.ijregi.2022.05.011

**Published:** 2022-06-04

**Authors:** Temesgen Getaneh, Getenet Dessie, Melaku Desta, Moges Agazhe Assemie, Addisu Alehegn Alemu, Getachew Tilaye Mihiret, Kumlachew Solomon Wondmu, Ayenew Negesse

**Affiliations:** a Department of Midwifery, College of Health Science, Debre Markos University, Debre Markos, Ethiopia; b Department of Nursing, College of Medicine and Health Science, Bahir Dar University, Bahir Dar, Ethiopia; c Department of Public Health, College of Health Science, Debre Markos University, Debre Markos, Ethiopia; d Department of Human Nutrition and Food Science, College of Health Science, Debre Markos University, Debre Markos, Ethiopia

**Keywords:** Early diagnosis, HIV, MTCT, PMTCT, Ethiopia

## Abstract

**Introduction:**

: Despite scaling up programmes to prevent vertical transmission of human immunodeficiency virus (HIV), over 400 children are infected every day globally. Guidelines recommend that all HIV-exposed infants should be tested within 4–6 weeks of birth. This meta-analysis aimed to explore concrete evidence on early diagnosis, pooled prevalence of HIV among HIV-exposed infants and its associated factors in Ethiopia following implementation of the Option B+ regime in 2013.

**Methods:**

: Electronic databases such as PubMed/MEDLINE, EMBASE and Cochrane Library were used to retrieve eligible articles. This meta-analysis was developed using the Preferred Reporting Items for Systematic Reviews and Meta-Analysis guidelines. Heterogeneity and publication bias were evaluated using Cochran's Q statistic quantified with inverse variance and Egger's statistical test. Random effect model meta-analysis was used to report the pooled estimate.

**Results:**

: The pooled estimate of early diagnosis and prevalence of mother-to-child transmission (MTCT) of HIV since implementation of the Option B+ regime in Ethiopia were 64.84% and 5.64%, respectively. No antenatal care [odds ratio (OR) 4.4], home delivery (OR 6.8), infant enrolment >6 weeks after birth (OR 2.2), mixed feeding (OR 4.1) and no antiretroviral prophylaxis after birth (OR 13.2) were associated with increased risk of MTCT.

**Conclusion:**

: This review of 19 studies from six regions of Ethiopia found that only two-thirds of HIV-exposed infants were tested at ≤6 weeks of age. Although there has been a major reduction in the prevalence of MTCT of HIV since implementation of the Option B+ region, the pooled prevalence of MTCT of HIV remains higher than the WHO target of 5% among breastfeeding participants. From the evidence obtained in the 19 studies included in this review and international MTCT practice, recommendations to minimize the high prevalence of MTCT of HIV include encouraging antenatal care follow-up and institutional delivery; enrolling HIV-exposed infants immediately after delivery or at least before 6 weeks of age; promotion of exclusive breastfeeding; improving adherence to antiretroviral therapy during antenatal care, labour/delivery and breastfeeding; and increasing the provision of antiretroviral prophylaxis at birth for exposed neonates. A national representative study of MTCT of HIV for all regions of Ethiopia is needed to obtain an updated view of this programme.

## Highlights

• In Ethiopia, only two-thirds of infants exposed to human immunodeficiency virus (HIV) are diagnosed early.
• The rate of mother-to-child transmission of HIV is still >5% since implementation of the Option B+ regime.
• Encouraging antenatal care and institutional delivery are very important.
• Promotion of exclusive breastfeeding and provision of antiretroviral prophylaxis at birth are recommended.

## Introduction

Human immunodeficiency virus (HIV) continues to be a major global public health threat, particularly in low- and middle-income countries. Globally, nearly 38 million people are living with HIV, 53% of whom are women (WHO, 2021). Sub-Saharan Africa is the hardest hit region in the world, accounting for more than two-thirds of all people living with HIV (UNAIDS, 2020). An estimated 20.7 million people are living with HIV in Eastern and Southern Africa, representing more than half (54%) of all people living with HIV (UNAIDS, 2021).

More than 1.7 million people living with HIV are children. Two-thirds of all children living with HIV (67%) are found in Eastern and Southern Africa. Nearly 90% of cases of mother-to-child transmission (MTCT) occur during pregnancy, labour/delivery or breastfeeding (UNICEF, 2016). In the absence of any intervention, MTCT can range up to 45% (WHO, 2010). However, increasing access to effective prevention of mother-to-child transmission (PMTCT) strategies, early HIV testing among exposed infants, provision of antiretroviral (ARV) prophylaxis at birth, and safe delivery practices can reduce MTCT of HIV to <5% (Ministry of Health, Federal Democratic Republic of Ethiopia, 2014).

In 2013, the World Health Organization (WHO) launched new guidelines for PMTCT called the ‘Option B+ regime’. The Option B+ regime is a simple option providing a combination of three ARV drugs/day for all HIV-positive mothers on the day of diagnosis regardless of their CD4 cell count (WHO, 2013). The Option B+ regime has benefits over previous options, including simplification of antiretroviral therapy (ART) to reduce errors and improve adherence, protection against MTCT in future pregnancies, and avoidance of stopping and starting ARV drugs (UNAIDS, 2016). More than 84% of pregnant women with HIV have received treatment to prevent MTCT and to protect their own health (WHO, 2017) in Ethiopia since implementation of the Option B+ regime in early 2013. The majority of HIV-infected women are on the Option B+ regime to remain healthy and to prevent transmission of HIV to their children (Ethiopian Federal Ministry of Health, 2016). Approximately 92% of pregnant women are on ART in Ethiopia (Kassaw et al., 2020b).

Despite scaling up programmes to prevent MTCT of HIV, over 400 children are infected with HIV every day globally. Nearly 30% of infected children will die by their first birthday, and half will die by their second birthday if left untreated (UNAIDS, 2016). Peak mortality occurs between 2 and 3 months of age as disease progression is more rapid in infants (Bourne et al., 2009). As such, it is essential to test HIV-exposed infants soon after birth. WHO recommends that all HIV-exposed infants should be tested within 4–6 weeks of birth. Although coverage has increased from 28% to 60%, it remains lower than the required level (UNAIDS, 2019).

In Ethiopia, early diagnosis of HIV among exposed infants ranges from 6.7% (Aga, 2017) in Oromia to 93.9% (Yitayew et al., 2019) in Amhara. The prevalence of MTCT of HIV following implementation of the Option B+ regime ranges from 1.3% (Deribessa et al., 2016) in Amhara and Tigray to 15.7% (Wudineh and Damtew, 2016) in Dire Dawa City. As such, this systematic review and meta-analysis aims to explore early diagnosis, pooled prevalence of HIV among HIV-exposed infants and its associated factors in Ethiopia following implementation of the Option B+ regime in 2013. In addition, it will investigate trends in effectiveness of the Option B+ regime.

## Methods

### Search strategy

Primary studies were searched using major electronic databases such as PubMed/MEDLINE, Cochrane Library, EMBASE, CINAHL, HINARI Portal and Google Scholar. Important key words, such as prevalence, incidence, burden, proportion, early diagnosis, HIV, acquired immunodeficiency virus, MTCT, PMTCT, HIV-exposed infant, neonate, baby, children, factors, predictors, risks and Ethiopia, with respective Medical Heading Subject terms combined with Boolean operators (OR and AND) were used to search primary studies on major electronic databases. In addition, local institutional repositories, libraries, research shelves, and cross-references of identified studies were checked to retrieve additional primary studies. The general search was conducted from 25 July 2021 to 5 August 2021. The Preferred Reporting Items for Systematic Reviews and Meta-Analyses protocol checklist guidelines (Maraolo, 2021) were followed during this meta-analysis (Appendix 1, see online supplementary material). Endnote Citation Manager Version X7 for Windows was used to remove duplicate studies.

### Eligible criteria

All studies conducted in Ethiopia on early diagnosis and prevalence of MTCT of HIV among exposed infants and associated factors since 2013 (following implementation of the Option B+ regime), that were reported in English, were included in this meta-analysis. No restrictions were imposed regarding study design or study setting. Studies that were not reported in English, qualitative studies, case reports, and studies that were not fully accessible were excluded.

### Outcome of the study

The primary outcome of this meta-analysis was to estimate the coverage of early testing of HIV among exposed infants. WHO recommends testing for HIV among exposed infants at ≤6 weeks of age using a deoxyribonucleic acid-polymerase chain reaction virology test. The second outcome of this meta-analysis was to estimate the pooled prevalence of MTCT of HIV among exposed infants since implementation of the Option B+ regime in Ethiopia. In addition, this meta-analysis aimed to explore the factors associated with MTCT of HIV in Ethiopia. The impacts of antenatal care (ANC) follow-up, home delivery, late enrolment of the infant to the service, mixed feeding for the first 6 months of life, and ARV prophylaxis at birth on MTCT of HIV among exposed infants were investigated.

### Quality assessment and data extraction

The Joanna Briggs Institute Meta-Analysis of Statistics Assessment and Review Instrument (JBI-MAStARI) adapted for observational studies was applied to examine the quality of the included studies (Schultz and Florence, 2007). The critical appraisal tool includes the following criteria: (1) appropriateness of sampling method and design; (2) appropriate sampling frame; (3) sample size adequacy; (4) standard measurement; (5) unbiased outcome measurement; (6) response rate; (7) confidence interval and subgroup analysis (if appropriate); and (8) appropriate study subjects. Two reviewers (TG and GD) evaluated the quality of articles independently. Those articles that scored at least five out of eight were considered as low risk and were included in this review (Appendix 2, see online supplementary material). Disagreement between reviewers was resolved through discussion and consensus. A third reviewer was involved to resolve inconsistencies between the two independent reviewers if necessary.

A data extraction form was creating using Excel (Microsoft Corp., Redmond, WA, USA), based on the JBI data extraction form, to extract data. Information included in the data extraction form included: name of first author; region (area); study year; study design; sample size; response rate; prevalence of early diagnosis; MTCT; and a 2 by 2 table with log odds ratio (OR) to show the effect of common factors such as ANC follow-up, home delivery, mixed feeding before 6 months of age, ARV prophylaxis at birth, and age of child at enrolment on MTCT of HIV. Any disagreement between the reviewers was resolved by discussion and consensus, and a third reviewer was involved if necessary.

### Data analysis

The extracted data were exported to STATA Version 14 (Stata Corp, College Station, TX, USA) for meta-analysis. The pooled prevalence of early diagnosis and overall burden of MTCT of HIV among exposed infants in Ethiopia since implementation of the Option B+ regime were determined using DerSimonian and Laird's random effects model. Cochran's Q statistic quantified using inverse variance (*I*^2^) was computed to assess the presence of heterogeneity in the included studies. Low, medium and high heterogeneity were considered at values of 25%, 50% and 75%, respectively (Huedo-Medina et al., 2006). In addition, Egger's statistical test was used to calculate publication bias for the included studies (Egger et al., 1997). Furthermore, subgroup analysis was undertaken using study area (region), study design and study year. Finally, Forest plots with 95% confidence intervals (CI) were used to report the results of this meta-analysis.

## Results

### Characteristics of individual studies

**Figure 1:**
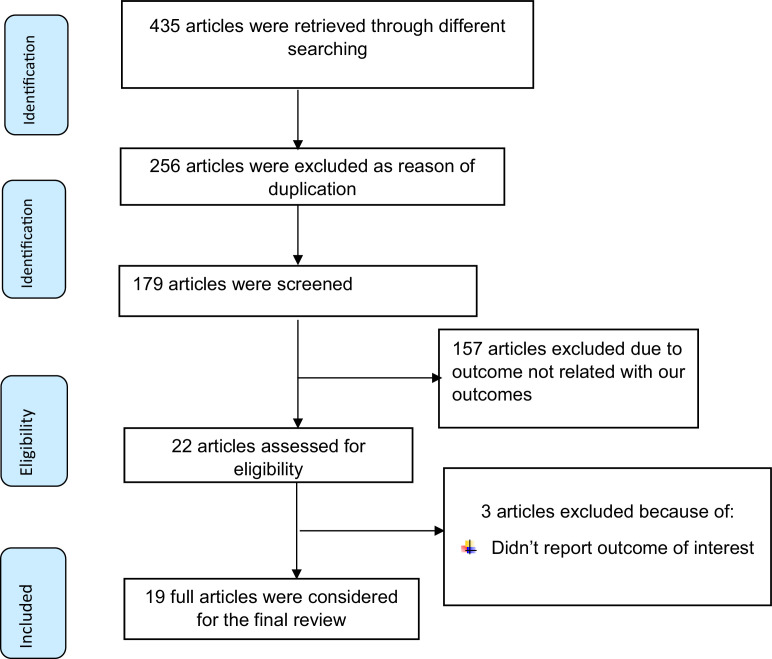
PRISMA flow diagram of included studies to estimate pooled prevalence of early diagnosis, and its outcome among human-immunodeficiency-virus-exposed infants following implementation of the Option B+ regime in Ethiopia.

In total, 435 studies were searched using major electronic databases. Of these, 413 studies were removed: 256 due to duplication and 157 due to their outcome not being related to the meta-analysis outcome. The full text of 22 studies was assessed for eligibility. Subsequently, three primary studies were excluded as they did not report the outcome of interest, so a total of 19 primary studies conducted in six regions of Ethiopia were included in this systematic review and meta-analysis (Figure 1).

Among the 19 studies included in this review, seven were from Amhara (Berhan et al., 2014; Deribessa et al., 2016; Moges et al., 2017; Tsehay, 2019; Yitayew et al., 2019; Kassaw et al., 2020a; Kassie et al., 2020), seven studies were from Oromia (Bayou et al., 2015; Olana et al., 2016; Aga, 2017; Mama et al., 2017; Obsa et al., 2018; Chaka et al., 2019; Debelew et al., 2020), two studies were from Tigray (Desta et al., 2019, Ebuy et al., 2020), and the remaining three studies were from Addis Ababa (Negash and Ehlers, 2016), South Nation Nationality and People (Yosef et al., 2020) and Dire Dawa (Wudineh and Damtew, 2016). The majority of the included studies were cohort studies (*n*=11) and eight studies had a cross-sectional design. All studies were conducted after implementation of the Option B+ regime in Ethiopia (2013), and were published from 2014 to 2020. In total, 6924 HIV-positive mothers with their exposed infants were included in the estimation of early diagnosis and its outcome (MTCT). Regarding MTCT, the lowest prevalence was reported in Amhara (1.37%) and the highest prevalence was reported in Dire Dawa (15.7%) (Table 1).

**Table 1:** Descriptive summary of studies included in this review of early diagnosis, mother-to-child transmission of human immunodeficiency virus among exposed infants in Ethiopia following implementation of the Option B+ regime.

Author	Study year	Study setting	Study design	Response rate (%)	Sample size	Prevalence (%)	JBI
Ebuy et al.	2017	Tigray	Cross-sectional	100	558	3.6	5
Deribessa et al.	2014	Amhara	Cross-sectional	100	658	1.37	8
Debelew et al.	2018	Oromo	Retrospective cohort	100	342	3.8	7
Bayou et al.	2014	Oromo	Cross-sectional	100	349	5.3	6
Tsehay et al.	2018	Amhara	Cross-sectional	100	477	5.8	8
Olana et al.	2014	Oromo	Retrospective cohort	100	624	4.3	7
Mesfin et al.	2015	Oromo	Retrospective cohort	100	225	5.3	5
Yosef et al.	2018	SNNPR	Cross-sectional	100	203	9	8
Kassie et al.	2018	Amhara	Retrospective cohort	99	239	5.5	5
Desta et al.	2016	Tigray	Cross-sectional	97	350	2.1	5
Mama et al.	2015	Oromo	Cross-sectional	100	126	7.7	8
Chaka et al.	2016	Oromo	Retrospective cohort	100	246	10	7
Berhan et al.	2013	Amhara	Retrospective cohort	100	434	10.1	6
Obsa et al.	2014	Oromo	Retrospective cohort	100	492	7.7	8
Negash et al.	2013	AA	Retrospective cohort	100	384	6	7
Moges et al.	2015	Amhara	Retrospective cohort	100	305	6.2	5
Kassaw et al.	2017	Amhara	Retrospective cohort	100	217	3.7	8
Wudineh et al.	2013	Dire Dawa	Retrospective cohort	100	382	15.7	5
Yitayew et al.	2017	Amhara	Cross-sectional	100	313	3.8	7

AA, Addis Ababa; JBI, Joanna Briggs Institute; SNNPR, South Nation, Nationality and People.

### Meta-analysis

**Figure 2:**
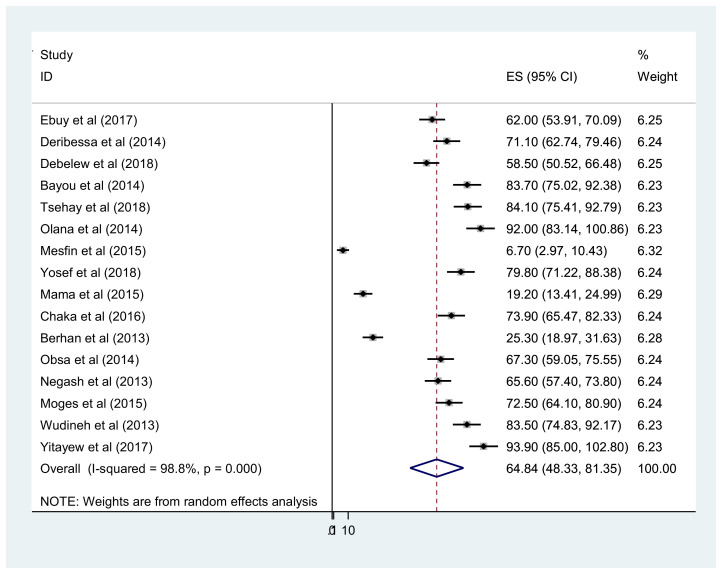
Forest plot of the pooled prevalence of early diagnosis of human immunodeficiency virus among exposed infants in Ethiopia.

**Figure 3:**
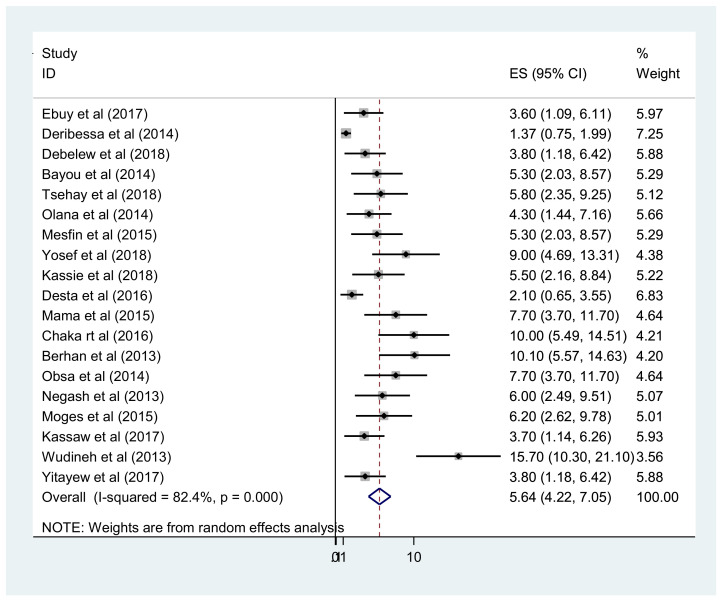
Forest plot of the pooled prevalence of mother-to-child transmission of human immunodeficiency virus among exposed infants in Ethiopia.

Overall, the pooled prevalence of early diagnosis of HIV-exposed infants in Ethiopia was 64.84% (95% CI 48–81) (Figure 2). In addition, the meta-analysis of 20 primary studies revealed that the pooled prevalence of MTCT after implementation of the Option B+ regime in Ethiopia was 5.64% (95% CI 4.22– 7.05). However, substantial heterogeneity was reported between studies (*I*^2^=82.4%; *P*<0.001) (Figure 3). Egger's statistical test excluded publication bias (*P*=0.432). To explore the possible sources of heterogeneity, subgroup analysis was undertaken as part of this meta-analysis based on study setting, study design and study period. The highest prevalence of MTCT was reported in Dire Dawa City and the lowest prevalence was observed in Tigray. In addition, the prevalence of MTCT of HIV decreased with time, with pooled MTCT of 6.6% among studies conducted from 2013 to 2015 and 4.6% among studies conducted from 2016 to 2018 (Table 2).

**Table 2:** Subgroup analysis showing pooled prevalence of mother-to-child transmission of human immunodeficiency virus among exposed infants following the implementation of the Option B+ regime in Ethiopia.

Subgroup		No of studies	Prevalence (95% CI)	Heterogeneity statistics	*I*^2^	*P*-value
Region	Amhara	7	4.8 (2.5,7.05)	31.9	82.3	<0.001
	Oromo	7	5.7 (4.2–7.3)	8.45	29	0.207
	Tigray	2	2.4 (1.2–3.7)	1.03	2.6	0.311
	Addis Ababa	1	6 (2.4,9.5)	–	–	–
	SNNPR	1	9 (4.6–13.3)	–	–	–
	Dire Dawa	1	15.7 (10.3–21)	–	–	–
Study year	2013–2015 inclusive	10	6.6 (3.9–9.2)	74.7	88	<0.001
	After 2015	10	4.6 (3.1–6.1)	20.4	60.8	0.009
Study design	Cross-sectional	8	4.2 (2.5–5.9)	33.86	79.3	<0.001
	Cohort	11	6.5 (4.8–8.2)	26.6	62.4	0.003

CI, confidence interval; SNNPR, South Nation, Nationality and People.

### Factors associated with MTCT

**Figure 4:**
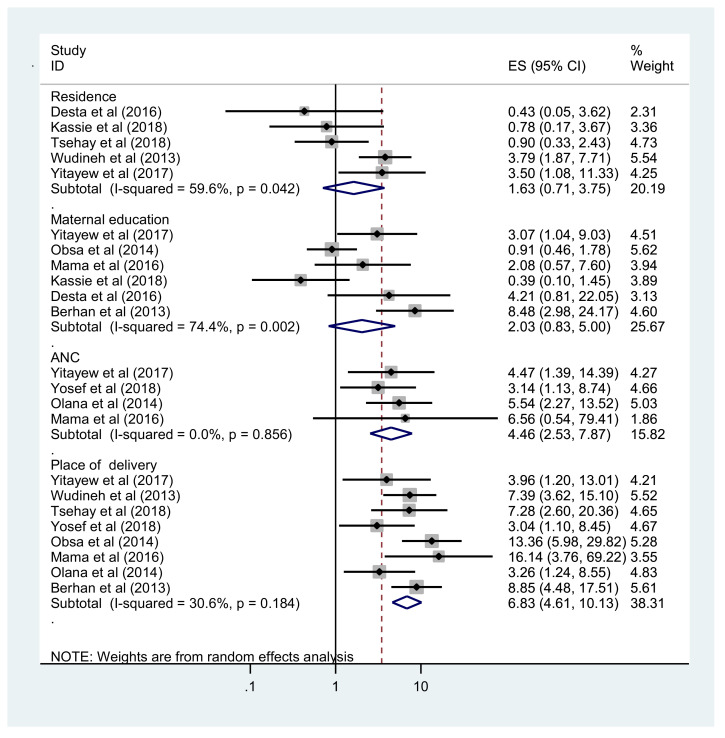
Forest plot showing the association between lack of formal maternal education, rural residence, lack of antenatal care (ANC) and home delivery, and mother-to-child transmission of human immunodeficiency virus in Ethiopia.

In addition to estimating early diagnosis and MTCT after implementation of the Option B+ regime, this meta-analysis also aimed to explore the pooled effect of common factors reported in the primary studies. Lack of ANC follow-up, home delivery, enrolment of infant at >6 weeks of age, mixed feeding before 6 months of age and absence of ART prophylaxis at birth were found to be significantly associated with MTCT. The prevalence of MTCT was 4.46 times higher among HIV-exposed infants whose mothers had not received ANC follow-up compared with their counterparts (OR 4.46, 95% CI 2.53–7.87). In addition, infants who were delivered at home were 6.83 times more likely to acquire HIV compared with infants born at a health facility (OR 6.83, 95% CI 4.61–10.13) (Figure 4).

**Figure 5:**
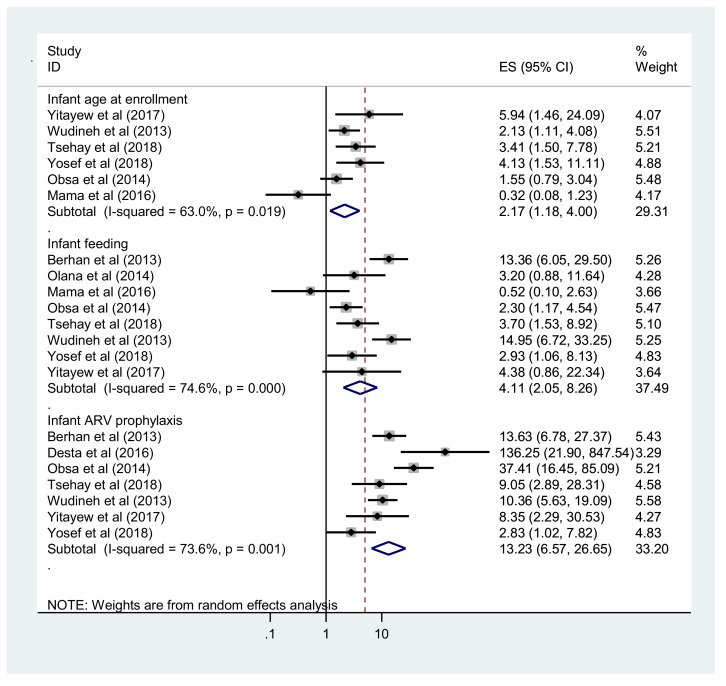
Forest plot showing the association between enrolment at >6 weeks of age, mixed feeding and no antiretroviral (ARV) prophylaxis at birth, and mother-to-child transmission of human immunodeficiency virus in Ethiopia.

Five studies were used to estimate the pooled effect of age of infant at enrolment on MTCT. HIV-exposed infants who had been enrolled at >6 weeks of age were 2.17 times more likely to be diagnosed with HIV compared with their counterparts (OR 2.17, 95% CI 1.18–4.0). Furthermore, the pooled analysis of eight primary studies showed that infants who received mixed feeding before 6 months of age were 4.1 times more likely to acquire HIV compared with infants who were exclusively breast fed for the first 6 months of life (OR 4.11, 95% CI 2.05–8.26). Moreover, infant ARV prophylaxis at birth, reported in seven primary studies, was found to be significantly associated with MTCT. The prevalence of MTCT was 13.23 times higher among HIV-exposed infants who had not received ARV prophylaxis at birth compared with their counterparts (OR 13.23, 95% CI 6.57–26.65) (Figure 5).

## Discussion

To the authors’ knowledge, this is the first systematic review and meta-analysis of studies on MTCT of HIV in Ethiopia since implementation of the Option B+ regime. The proportion of infants who received timely testing for an early diagnosis of HIV was 64.8% (95% CI 43–81). This indicates that only two-thirds of HIV-exposed infants were tested at ≤6 weeks of age in Ethiopia. This study highlights the reduction in MTCT of HIV among infants since implementation of the Option B+ regimen, although the reduction is suboptimal (not yet <5%). A study conducted in Cameroon showed similar results (Tejiokem et al., 2011). This finding is higher than the results reported from studies in Tanzania (Nuwagaba-Biribonwoha et al., 2010) and Kenya (Hassan et al., 2012), but lower than the results reported from Lesotho (Gill et al., 2017).

The pooled prevalence of MTCT (to 18 months) of HIV following implementation of the Option B+ regime was 5.64% (95% CI 4.22–7.05). This is much lower than the MTCT rate before implementation of the Option B+ regime in Ethiopia, which was 14.32%. This may be because the Option B+ regimen is better than previous options for the prevention of MTCT. The Option B+ regime involves three ARV drugs/day for pregnant and breastfeeding women at the time of HIV diagnosis, regardless of their CD4 count or clinical stage. However, the pooled prevalence rate is still higher than the WHO-desired target for MTCT among breastfeeding mothers. This could be due to suboptimal adherence to the Option B+ regime. Therefore, interventions such as promoting disclosure of status, social and financial support, counselling on side effects, increasing awareness about PMTCT, and preventing stigma/discrimation among patients with HIV are recommended to improve the level of adherence to the Option B+ regime in Ethiopia, and then to decrease MTCT.

The pooled prevalence of MTCT in this study was higher than that reported in a systematic review from China (2.1%) (Zeng et al., 2016). This could be due to sociodemographic and health system differences. It was also higher than the rate reported from Tanzania (Gamell et al., 2017). In addition, the present result is lower than that reported in a meta-analysis from East Africa (Belachew et al., 2020). This may be because the meta-analysis conducted in East Africa included studies from countries with higher HIV burdens and studies conducted before 2013 (before implementation of the Option B+ regime). Studies conducted in Kenya (Sirengo et al., 2014), South Africa (Rollins et al., 2002) and Nigeria (Ogunbosi et al., 2011) reported higher prevalence of MTCT of HIV than the present meta-analysis. The findings of the present meta-analysis are in line with studies reported from Eritrea (Teclebirhan et al., 2009) and Uganda (Kahungu et al., 2018).

Regarding subgroup analysis, the highest prevalence of MTCT was observed in Dire Dawa City and the lowest prevalence was found in Tigray. This suggests that there is a difference between regions in terms of the priority given to PMTCT, maternal and child health expansion, and the prevalence of HIV among the population. Furthermore, the prevalence of MTCT of HIV among exposed infants decreased, from 6.6% among studies reported before 2016 (after implementation of the Option B+ regime) compared with 4.6% among studies conducted after 2016. This indicated improvement in Option B+ service coverage and utilization over a period of time, and better adoption of the guidelines across the country.

ANC follow-up was found to be an important factor associated with MTCT of HIV. The OR of MTCT of HIV was higher among infants whose mothers did not receive ANC follow-up. This finding is supported by evidence from Kenya (Turan et al., 2015). The possible reason may be that ANC follow-up enables early screening and enrolment of mothers and their newborns into the PMTCT service, increases provision and use of institutional delivery, affects decisions regarding feeding options, and improves the likelihood of accessing ARV prophylaxis at birth, with treatment enrolment if required. In addition, supporting drug adherence during ANC, labour/delivery and breastfeeding is a very important step.

Home delivery also increased the OR of MTCT. The prevalence of MTCT was higher among infants born at home compared with those born at health institutions. This finding is in line with the findings of a systematic review from East Africa (Belachew et al., 2020), and studies conducted in Nigeria (Ogunbosi et al., 2011) and Zimbabwe (Ngwende et al., 2013). This could be because infants born at home do not receive PMTCT services, such as testing and ARV prophylaxis if positive; safe delivery practices, such as early cord cutting; and prelacteal breast feeding; this increases their risk of vertical transmission (Becquet et al., 2008; Boer et al., 2010; Ashiono et al., 2017).

The OR of MTCT of HIV was twice as high among newborns who had been enrolled in PMTCT services at >6 weeks of age. This finding is in line with a study conducted in Kenya (Okoko et al., 2017). This is because early enrolment of exposed infants to PMTCT services promotes early ARV prophylaxis, which leads to viral suppression and a reduction in viral load.

HIV-exposed infants who received mixed feeding for the first 6 months of life were four times more likely to acquire HIV infection compared with infants who were exclusively breast fed. This finding was supported by the results of a systematic review in East Africa (Belachew et al., 2020), and a study conducted in Zimbabwe (Ngwende et al., 2013). Antigens in non-breast milk are thought to cause inflammation in the infant gut, making it more vulnerable to HIV infection (Wise, 2001).

Moreover, the OR of MTCT of HIV was higher among HIV-exposed infants who had not received ARV prophylaxis at birth. This finding is consistent with the findings of studies from Brazil (De Lemos et al., 2013) and Uganda (Guay et al., 1999). A study conducted in India found that ARV prophylaxis administered to infants reduced the MTCT rate seven-fold (Potty et al., 2019). This is because ARV prophylaxis aims to prevent HIV infection in infants by viral suppression (Ayouba et al., 2003).

### Limitations

To the authors’ knowledge, this is the first meta-analysis in Ethiopia to explore the pooled prevalence of early diagnosis of HIV among exposed infants, its outcome and associated factors since implementation of the Option B+ regime. Limitations of this review include observed heterogeneity between primary studies, although subgroup analysis was reported; the use of retrospective or recorded data, which may be affected by incompleteness and inaccuracy; and the absence of primary studies from some regions of Ethiopia. All of the eligible studies were conducted in six regions of Ethiopia. Substantial heterogeneity between the primary studies, including the estimated effect size, was also observed, although subgroup analysis was reported. Finally, this systematic review included cross-sectional studies, which made it difficult to predict the cause–effect relationship (even after the cause–effect relationship was checked following exclusion of cross-sectional studies).

## Conclusion

This review of 19 studies from six regions of Ethiopia found that only two-thirds of HIV-exposed infants were tested at ≤6 weeks of age. Although there has been a major reduction in the prevalence of MTCT of HIV since implementation of the Option B+ regime, the pooled prevalence of MTCT of HIV remains higher than the WHO target of 5% among breastfeeding participants. From the evidence obtained in the 19 studies included in this review and international MTCT practice, recommendations to minimize the high prevalence of MTCT of HIV include encouraging ANC follow-up and institutional delivery; enrolling HIV-exposed infants immediately after delivery or at least before 6 weeks of age; promotion of exclusive breastfeeding; improving adherence to ART during ANC, labour/delivery and breastfeeding; and increasing the provision of ARV prophylaxis at birth for exposed neonates. A national representative study of MTCT of HIV for all regions of Ethiopia is needed to obtain an updated view of this programme.

## Funding

None.

## Ethical approval

Not required.

## Author contributions

TG developed the protocol and was involved in the design, selection of study, data extraction, statistical analysis and developing the initial drafts of the manuscript. TG, GD, MD and MAA were involved in quality assessment. TG, GD, MD, MAA, AA, GTM, KSW and AN prepared and revised subsequent drafts, and prepared the final draft of the manuscript. All authors read and approved the final draft of the manuscript.

## Conflict of interest statement

None declared.

## Appendix Supplementary materials

Image, application 1

Image, application 2
